# Low level of knowledge about cervical cancer among Ethiopian women: a systematic review and meta-analysis

**DOI:** 10.1186/s13027-021-00350-x

**Published:** 2021-02-10

**Authors:** Awoke Derbie, Daniel Mekonnen, Eyaya Misgan, Yihun Mulugeta Alemu, Yimtubezinash Woldeamanuel, Tamrat Abebe

**Affiliations:** 1 Department of Medical Microbiology, College of Medicine and Health Sciences, Bahir Dar University, Bahir Dar, Ethiopia; 2 Centre for Innovative Drug Development and Therapeutic Trials for Africa (CDT-Africa), Addis Ababa University, Addis Ababa, Ethiopia; 3 Department of Health Biotechnology, Biotechnology Research Institute, Bahir Dar University, Bahir Dar, Ethiopia; 4 Department of Gynecology and Obstetrics, College of Medicine and Health Sciences, Bahir Dar University, Bahir Dar, Ethiopia; 5 Departent of Epidemiology and Biostatistics, College of Medicine and Health Sciences, Bahir Dar University, Bahir Dar, Ethiopia; 6 Department of Medical Microbiology, Immunology and Parasitology, School of Medicine, College of Health Sciences, Addis Ababa University, Addis Ababa, Ethiopia

**Keywords:** Knowledge, Cervical cancer, Ethiopia

## Abstract

**Introduction:**

Cervical cancer is one of the leading causes of malignancies among women in Ethiopia. Knowing the disease could empower women to make an informed decision regarding participation with cervical cancer prevention strategies. There is scarcity of compiled data in the field. Therefore, this systematic review aimed to provide an overview of knowledge about cervical cancer among Ethiopian women.

**Methods:**

We conducted a systematic review of peer-reviewed articles on the knowledge of cervical cancer. Articles were systematically searched using comprehensive search strings from PubMed/Medline, SCOPUS, and grey literature from Google Scholar. Two reviewers assessed study eligibility, extracted data, and the risk of bias independently. Meta-analysis was performed using STATA v 14 to pool the overall knowledge of the women about cervical cancer.

**Results:**

We included 26 articles published between 2013 and 2020 covering a total of 14,549 participants. All the included articles had good methodological quality. The proportion of participants who had heard of cervical cancer varied from 4.6 to 87.7% with the pooled estimate at 56% (95% CI: 47–66). Similarly, the proportion of participants who knew that HPV is the main cause of cervical cancer lied between 0 and 49.7% with the pooled result at 21% (95% CI: 13–30). Likewise, the pooled prevalence to identify at least one risk factor, one symptom of cervical cancer and ever heard of cervical cancer screening was gauged at 52% (95% CI: 39–64), 43% (95% CI: 26–60), and 39% (95% CI: 24–55), respectively. The overall pooled prevalence of good knowledge about cervical cancer was at 43% (95% CI: 33–53). On top of this, the prevalence of previous screening practice among the participants was at 14% (95% CI: 9–20).

**Conclusions:**

Knowledge about cervical cancer among Ethiopian women is quite poor. Therefore, health education to provide sufficient and unbiased information about HPV and cervical cancer in general is required to the public.

## Background

Cervical cancer ranks the fourth most frequent cancer among women and is the 2nd most common female cancer in the age group between 15 to 44 years worldwide. Every year over 527,624 women are diagnosed with the case and about 265,672 die from the disease globally [1]. In Ethiopia women aged 15 years and older, who are at risk of developing cervical cancer, are close to 30 million. Based on the 2018 World Health Organization (WHO) report, of all form of cancers in Ethiopia, new cervical cancer cases and death shared 6294 (10.6%) and 4884 (11.6%), respectively [2]. Cervical cancer is the leading cause of cancer-related mortality among women and almost nine out of ten cervical cancer deaths disproportionately occur in developing countries [3–9].

More than 99% of cervical cancer is associated with genital infection with certain types (High-Risk) Human papillomavirus (HPV), which is the most common viral sexually transmitted infection (STI) globally [10, 11] that most women are experiencing soon after they become sexually active [12]. Persistent infection with HR-HPV is the primary cause of cervical cancer [13, 14]. There are identified factors that increase the risk of cervical cancer. Among them some are multiple numbers of sexual partners, early age at first sexual intercourse, presence of STIs, use of hormonal contraceptives for a long time, multiple parity, elderly age and smoking behavior [15]. Moreover, the health policy on cancer, capacity of the health system, socio-economic and cultural factors and awareness and knowledge about the disease was found linked with cervical cancer related morbidity and mortality [15, 16].

Cervical cancer is curable if detected at its early stage. Premature detection is valuable as early lesions develop slowly preceding cancer, typically over a period of 10 years [17]. These precursor lesions, are detectable by a variety of methods; to mention cytology (Pap smear), HPV-based tests and visual inspection with acetic acid (VIA) are common [18]. In the Ethiopian context, visual inspection of the cervix with acetic acid is routinely used and is giving promising results in terms of reducing morbidity and mortality [15].

Several evidences showed that, knowledge and awareness of cervical cancer among African women, in general, is very poor while mortality is very high [16]. Women’s knowledge of cervical cancer would impact the uptake of cervical cancer prevention activities, like avoiding risk factors, to get regular screening and vaccination. Hence, there is a need to have compiled data on women’s knowledge about cervical cancer as input for intervention which is otherwise quite limited in Ethiopia even if there are fragmented reports. Therefore, this review was conducted aimed at assessing the overall knowledge of Ethiopian women about cervical cancer.

### Review question

This systematic review addresses the following question; how is the knowledge of Ethiopian women on cervical cancer?

### Objective

The main aim of this review was to describe the knowledge and awareness of Ethiopian women on cervical cancer.

## Methods

### Protocol registration

Following Preferred Reporting Items for Systematic Reviews and Meta-Analysis (PRISMA) guideline, the review protocol was registered at International Prospective Register of Systematic Reviews (PROSPERO) with a registration number of CRD42020219684.

### Eligibility criteria

Studies were selected based on the following criterion: *Study design*: descriptive studies that reported the knowledge of women on cervical cancer. *Participants*: women of any age group from the general population. Studies that employed health professionals were not considered in this review as a significant proportion of health care workers are assumed to be knowledgeable about cervical cancer [19]. *Setting*: we included studies with the outcome of interest reported in Ethiopia. *Language and publication*: We included peer-reviewed published articles and unpublished preprints written in the English language.

### Information sources and search strategy

This review was done following PRISMA [20]. A computerized systematic strategy was adopted to search for articles in PubMed/Medline and SCOPUS. The last search was conducted on 29 September 2020. Manual search from Google Scholar and Google databases was also carried out for grey literature. The search terms were developed in line with the Medical Subject Headings (MeSH) thesaurus using a combination of the big ideas (or ‘key terms’) which derived from the research question. The reference lists of retrieved articles were probed (forward and back ward searching) to identify articles that were not retrieved from databases manual search. The first two authors; AD and DM searched the articles independently.

The domains of the search terms were: ‘knowledge’, ‘awareness’, ‘cervical cancer’ ‘Ethiopia’. We combined these terms using the Boolean operator “OR”, and “AND” accordingly. The full search strategy for the two databases is annexed in Supplement 1.

### Study selection

Studies that have reported the knowledge of Ethiopian women on cervical cancer were included regardless of their year of publication. Searched articles were directly imported and handled using EndNote X5 citation manager (Thomson Reuters, New York, USA). Based on the PRISMA procedure, duplicated articles were excluded and the titles and abstracts of the remaining papers were screened sequentially for inclusion in full-text evaluation by the first two authors. Differences between the reviewers were resolved through discussion.

### Data collection process and data items

The extracted data items include name of the first author, publication year, mean/median age of the study participants, sample size, variables related to the knowledge of the study subjects about cervical cancer. The data were extracted from the included articles using piloted excel data extraction sheet developed by the first author.

### Methodological quality appraisal of the included studies

The validity and methodological quality of all included studies were assessed using Joanna Briggs Institute Critical Appraisal Checklist for systematic review [21]. The tool consists of eight criteria that were checked as ‘yes’, ‘no’, ‘unclear’ or ‘not applicable’. After carefully evaluating the included articles against each criterion, studies were finally classified in to three groups; a study that fulfilled > 80% of the criteria was considered as ‘good quality’. Similarly a study that scored 50–80 and < 50% were rated as ‘fair’ and ‘poor’ quality, respectively. Fortunately, all the included studies scored > 80 and judged methodologically good.

### Data synthesis

Descriptive statistics, such as: simple counts, ranges and percentages were used to present the synthesized data. A systematic narrative synthesis was provided in which summary results were presented using text and table. To pool the overall knowledge of the women about cervical cancer meta-analysis was performed using STATA v 14 (Stata Corp. College Station, TX, USA) using a random effect model. The heterogeneity of the included studies was assessed using the *I*^2^ test and *I*^2^ = > 50% considered as high heterogeneity among the results of the included studies.

### Operational definition

#### Knowledge

The included studies used a series of different number of items to measure knowledge. The level of knowledge was measured using items related to risk factors, signs and symptoms, treatment options, and prevention methods and early detection measures of cervical cancer. Studies used the cumulative mean score of the participants about cervical cancer to measure their knowledge. Based on this, they labeled as poor knowledge for those who had scored less than the mean and good knowledge for those who had scored greater than or equal to the mean value.

## Results

### Search results

**Fig. 1:**
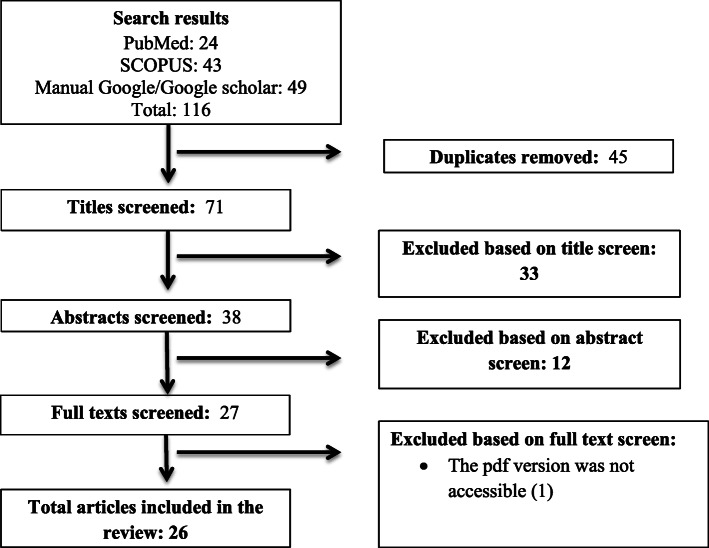
The PRISMA flow diagram of literature selection

From the computerized systematically searched databases and other sources, a total of 116 articles were retrieved and sequentially screened for inclusion in the analysis as depicted in PRISMA flow chart (Fig. 1) [20]. Twenty-six articles met our inclusion criteria and were included in the systematic review and meta-analysis.

### Characteristics of the included studies

The characteristic of the included studies is summarized in (Table 1). All included studies were published in the period of 2013 and 2020 and used a cross-sectional study design to describe the knowledge of women on cervical cancer. These articles used questionnaires adapted from previously published similar articles to generate data.

**Table 1:** Descriptive summary of studies included in the systematic review

Author, year	Study area	Region	Study subjects					
			Sample size	Include age groups	Mean age, SD^b^	Urban residence, n (%)	No formal education, n (%)	Marital status, married, n (%)
Tesfaye, 2017	Gondar	Amhara	267	18–24	20.6 ± 1.2	223 (83.5)	nr	nr
Ruddies, 2020	Butajjira	Oromia	342	30–49	35.5 ± 5.6)	34 (9.4)	217 (63.6)	325 (95.3)
Getachew,2020	Addis Ababa	AA	520	^a^nr	27.7 ± 5.5	nr	77 (14.8)	369 (71)
Bayu, 2016	Mekelle	Tigri	1186	nr	31.3 ± 9.3	nr	145 (12.2)	768 (64.8)
Nigussie, 2019	Jimma	Oromia	737	30–49	36.6 ± 5.3	nr	166 (22.5)	610 (82.8)
Kasim, 2020	Sidama zone	Sidama	506		37 ± 5.3	nr	185 (36.6)	468 (92.5)
Getahun, 2013	Gondar	Amhara	633	> 15	31 ± 11.3	nr	118 (18.8)	281 (44.6)
Erku, 2017	Gondar	Amhara	302	> 17	33.72 ± 9.7	211 (69.9)	33 (10.9%)	179 (59.3)
Geremew, 2018	Finote Selam	Amhara	1137	30–49	37.4 + 5.7	nr	513 (45.1)	755 (66.6)
Gebregziabher, 2019	Aksum	Tigri	344	19–30	23.7 ± 2.8	261 (75.9)	0	28 (8.1)
Mitiku, 2016	Dessie	Amhara	620	15–49		nr	262 (42.3)	382 (61.6)
Bulto, 2019	West Shoa	Oromia	423	> = 18	34.7 ± 7.2	346 (81.8)	109 (25.8)	199 (47)
Mengesha, 2019	Gondar	Amhara	770	24–32		579 (75.2)	82 (10.6)	420 (54.5)
Tsegaye, 2018	Hawassa	SNNP	380	18–26	21.5	Nr		12 (3.2)
Shiferaw,2016	Multicenter		432	21–49	31.4 ± 4.8	367 (85)	143 (33.1)	171 (39.6)
Saleem, 2019	Jimma	Oromia	154	nr	45.2 ± 11.2	59 (38)	133 (86.4)	107 (72.29)
Mruts, 2018	Debre Berhan	Amhara	584	17–38	20.5 ± 1.8	214 (37.5)	nr	23 (4)
Tefera, 2016	Bale Zone	Oromia	363	18–49	28 ± 7.5	nr	44 (12.2)	268 (73.8)
Aweke, 2017	Hossana	SNNP	583	18–48	28 ± 6.83	nr	74 (12.7)	366 (62.8)
Tilahun, 2019	Nekemte	Oromia	805	17–26	22.8	nr	nr	36 (4.5)
Kasa, 2018	Finote Selam	Amhara	735	17–88	30.8 ± 9.8	nr	111 (15.1)	290 (39.5)
Tekle, 2020	Wolaita Zone	SNNP	516	30–49	36.8 ± 5	345 (66.9)	110 (21.3)	309 (59.9)
Segni, 2017	Addis Ababa	AA	508	21–64		nr	211 (46.5)	454 (89.4)
Indracanti, 2018	Gondar	Amhara	283	17–30	20.86 ± 1.9	144 (50.9)	nr	21 (7.4)
Chaka, 2018	Gamo Gofa	SNNP	799	18–69	30	nr	347 (43.4)	596 (74.6
Tefera, 2017	Dessie	Amhara	620	15–49	Nr	nr	262 (42.3)	382 (61.6)

AA: Addis Ababa, ^a^nr: not reported, ^b^*SD* Standard Deviation

Most of the studies were reported from the four regions of the country; Tigri, Amhara, Oromia and Southern Nations, Nationalities and Peoples regions (SNNP). Two articles [22, 23] from Addis Ababa and one article [24] was from Sidama regional state. The number of participants in each included article varied from 154 [25] to 1186 [26]. Overall, this review contains reports of 14,549 study participants. The reported age group and the mean age of the participants were variable. However, all participants were in the age group of 15–88 years and the mean age ranges between 20.5–38 years. The majorities of the participants were married and had no formal education. With regard to the HIV status of the study participants, two of the included studies employed all HIV-positive women [27, 28]. The other three studies, [24–26] reported the proportion of HIV positive women at 5.5%. 8.7 and 22%, respectively. The remaining articles had no information about the HIV status of the study participants.

### Knowledge of women on cervical cancer

The pooled prevalence of women who had ever heard about cervical cancer in Ethiopia was 56% (95% CI: 47–66). Largely, mass-media (television/radio) mentioned as the main source of information about the disease. Sadly, the pooled proportion of knowing HPV as the main cause of cervical cancer was at 21% (95% CI: 13–30) which is very low. Similarly, the pooled prevalence of identifying at least one risk factor, one symptom of cervical cancer and ever heard of cervical cancer screening was at 52% (95% CI: 39–64), 43% (95% CI: 26–60) and 39%(95% CI:24–55), respectively. However, heterogeneity was very high (*I*^2^ = > 98%) and the result must be interpreted with caution (Table 2).

**Table 2:** Pooled estimates for some of the selected variables on knowledge about cervical cancer

Variables	Pooled estimate (%)	95%CI	I-squared (%)	Number of included studies in the analysis
Ever heard of CC	56	47–66	99.4	25
Know that HPV is the main cause of CC	21	13–30	99.2	11
Identified at least one risk factor of CC^a^	52	39–64	99.5	17
Identified at least one symptom of CC^b^	43	26–60	99.5	10
CC is treatable	46	34–59	99.2	12
Identified at least one treatment option of CC^c^	62	38–85	99.6	6
CC is preventable	55	46–63	98.1	11
Ever heard of CC screening	39	24–55	99.7	13

^a^Example: Early onset of sexual activity, having multiple sexual partner, multi-parity, STI, prolonged use of oral contraceptive, positive family history for cervical cancer

^b^Example: Vaginal discharge, intermenstrual bleeding, bleeding between periods, bleeding after intercourse, post-menopausal bleeding

^c^Chemotherapy, radiation, surgery

### Overall knowledge about cervical cancer

**Fig. 2:**
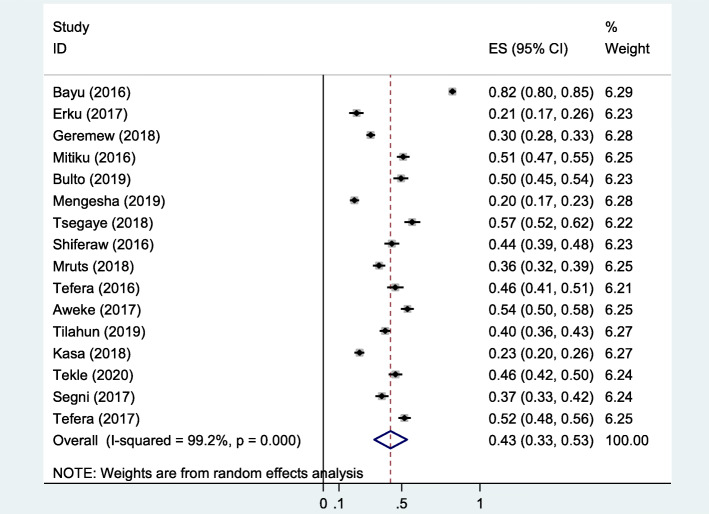
Frost plot showing the overall knowledge of cervical cancer in Ethiopia

The reported good knowledge level of the participants about cervical cancer by the included studies ranges from 19.9% [29] to 82.5% [26] (Table 3). The pooled overall knowledge was at 43% (95% CI: 33–53) (Fig. 2).

**Table 3:** The proportion of good knowledge reported by the included studies about cervical in Ethiopia

Author	Year of publication	Knowledgeable about CC	
		n	%
Ruddies et al.	2020	nr^a^	
Bayu et al.	2016	979	82.5
Getahun et al.	2013	195	31
Erku et al.	2017	64	21.2
Geremew et al.	2018	341	30.3
Mitiku et al.	2016	316	51
Bulto et al.	2019	210	49.6
Mengesha et al.	2019	153	19.9
Tsegaye et al.	2018	216	56.8
Shiferaw et al.	2016	135	43.8
Mruts et al.	2018	195	35.6
Tefera et al.	2016	121	46
Aweke et al.	2017	313	53.7
Tilahun et al.	2019	318	39.5
Kasa et al.	2018	170	23.1
Tekle et al.	2020	154	46.1
Segni et al.	2017	190	37.4
Indracanti et al.	2018	nr	
Chaka et al.	2018	nr	
Tefera et al.	2017	322	51.9

^a^*nr* Not reported

On top of this, thirteen articles had reported about cervical cancer screening history of the study participants and the pooled prevalence of previous screening history was at 14% (95% CI: 9–20, *I*^*2*^ = 98.6%).

## Discussion

The incidence and prevalence of cervical cancer are higher in the developing world due to several reasons such as lack of awareness regarding HPV infection, absence of organized cervical cancer education, screening and vaccination programs. Knowledge about the disease provides a vital prospect to undertake comprehensive prevention and control strategies in the community. Therefore, this particular review summarized articles containing information about the knowledge of Ethiopian women about cervical cancer as an entry point for the prevention and control of the disease.

In this review, about 56% (95% CI: 47–66) of women had ever heard about cervical cancer. Mass-media (television/radio) was the most frequently reported source of information about the disease. The proportion of good knowledge by the included studies ranges from 19.9% [29] to 82.5% [26] and the pooled overall good knowledge about cervical cancer gauged at 43% (95% CI: 33–53) which is considered as very low. Hence, education about HPV, cervical cancer and associated prevention methods must be strenghned and has to be integrated with sexual and reproductive health, antenatal health system in health facilities, universities and various associations. A similar review article by Perlman et al. showed that the levels of knowledge on cervical cancer and HPV were consistently low in Sub-Sharan Africa [30]. Similarly, a review by Cunningham and his colleague stated that there is a broad knowledge gap concerning HPV and cervical cancer among African women [31]. A low level of knowledge of cervical cancer among Indian women is also reported by Husain et al. [32]. In contrast, overall good knowledge of cervical cancer was reached as high as 78.6% in Saudi Arabia [33] which might be because of their better socio-economic status than Ethiopia.

In our review the pooled proportion of knowing HPV as the main cause of cervical cancer was at 21% (95% CI: 13–30). A global-based review by Klug and colleagues on knowledge about infection with HPV showed that the knowledge of the general public about HPV infection was poor and the proportion of participants who even had heard of the term HPV varied from 13 to 93% [34]. A similar review article in Iran by Taebi et al. corroborated with our report of a low level of knowledge about HPV [35]. Taken together, women’s knowledge was very low across different continents. Therefore, increasing the knowledge of women about HPV could empower them to make an informed decision regarding participation with cervical cancer prevention approaches [36].

The pooled prevalence of identifying at least one risk factor, one symptom of cervical cancer and ever heard of cervical cancer screening was at 52% (95% CI: 39–64), 43% (95% CI: 26–60) and 39%(95% CI:24–55), respectively. These figures imply that there is a relatively low level of information about cervical cancer among Ethiopian women. This review justifies an urgent need for government and non-government organizations, health-care providers and other stakeholders to join hands together to educate women, create massive social awareness to reduce the risk of having cervical cancer in the community [32, 33].

Avoiding or minimizing risk factors and early detection of precancerous cervical lesions are key to reduce the incidence and prevalence of cervical cancer. In this review the pooled proportion of women who knew that cervical cancer can be preventable was at 55% (95% CI: 46–63) (Table 2) which is suboptimal. Cervical cancer is potentially preventable hence there is a need to diversify strategies to reach women though different communication channels. Furthermore, approaches to increase uptake of screening practice and introduction of the latest point of care screening tools to the lowest level health tier system can have a profound impact in bringing awareness and raising their knowledge [37].

One of the prominent means of cervical cancer prevention is screening. In the present study, thirteen articles had reported about cervical cancer screening history of the study participants and the pooled prevalence of previous screening practice was at 14% (95% CI: 9–20, *I*^*2*^ = 98.6%). The reported poor knowledge (43%) about cervical cancer might be linked with this poor screening uptake. It has been reported that the knowledge of women on cervical cancer was a determinant factor for cervical cancer screening uptake [38]. Further, the majority of women in this review had no formal education. Literacy was also reported to be an independently associated factor with cervical cancer screening uptake [39]. According to a review by Desalegn et al, the pooled prevalence of cervical cancer screening uptake among HIV-positive women in Ethiopia was about 18% [38] which is slightly higher than our report. In Ethiopia, HIV-positive women had better priority for cervical cancer screening than the general public [40] which might explain the relativly higher screening uptake of this review. Although our review didn’t put attempt to find out factors that limit the uptake of cervical cancer screening in Ethiopia, Desalegn et al. review showed that educational status of women, knowledge of women on cervical cancer, and perceived susceptibility were significantly associated with cervical cancer screening uptake [38]. A similar review by Lim and colleagues aimed at identifying barriers to utilize cervical cancer screening in Sub Sahara Africa showed that low level of awareness about cervical cancer prevention services, embarrassment and possible violation of privacy, lack of spousal support, societal stigmatization, cost of accessing services and health service factors like proximity to the facility, facility navigation, waiting time and health care personnel attitude were the main factors the limit women to uptake screening [41].

Taken together, there is a need to improve the existing national strategies of cervical cancer screening [40] uptake. An effective screening and integrated sustainable education programs about the general picture of cervical cancer can help to increase public awareness and eventually can lead to a significant reduction in morbidity and mortality associated with the disease [42, 43].

### Strength and limitations

To the best of our knowledge, this systematic review reported the latest summarized finding of knowledge of women on cervical cancer in the Ethiopian context. However, our finding should be interpreted with caution due to some drawbacks; all the studies were reported from the four regions and the Addis Ababa city administration. The absence of data from the rest of the regions in the country might bias the overall national estimate in to these regions. The other snare of this review is the presence of high heterogeneity between the included articles.

## Conclusion

The result of this review showed that the overall knowledge about cervical cancer was found to be poor in Ethiopia that needs an improvement as the disease is increasing. This study urges the stake holder’s engagement in the battle like the provision of organized education at various levels from school to house to house using different mainstream Medias and associations.

## Supplementary Information

**Additional file 1:** Search strategy.

## Data Availability

All the generated data in this review are included in the manuscript. The original data can be obtained from the principal investigator upon request.

## Abbreviations

HPV: Humanpapillom Virus
HIV: Human Immunodeficiency virus
Pap: Papaniculo test
VIA: Visual Inspection with Acetic acid
WHO: World Health Organization
PRISMA: Preferred Reporting Items for Systematic Reviews and Meta-Analysis
SNNP: Southern Nations, Nationalities and Peoples regions
