# Supplementary material for: Low level of knowledge about cervical cancer among Ethiopian women: a systematic review and meta-analysis
Source: Infect Agent Cancer. 2021 Feb 10;16:11. doi: 10.1186/s13027-021-00350-x (PMC7876815; doi:10.1186/s13027-021-00350-x)
Supplement: Supplementary file 1 — Additional file 1: Search strategy. [file 13027_2021_350_MOESM1_ESM.docx]

Supplement 1: Search strategy

PubMed: ((("Patient Medication Knowledge"[Mesh] OR "Knowledge Bases"[Mesh] OR "Health Knowledge, Attitudes, Practice"[Mesh] OR "Metacognition"[Mesh] OR "Knowledge"[Mesh]) OR "Awareness"[Mesh]) AND "Uterine Cervical Neoplasms"[Mesh]) AND "Ethiopia"[Mesh]

Scopus: -ABS-KEY ( knowledge ) AND TITLE-ABS-KEY ( awareness ) AND TITLE-ABS-KEY ( cancer ) ) AND ( LIMIT-TO ( AFFILCOUNTRY , "Ethiopia" ) ) AND ( LIMIT-TO ( DOCTYPE , "ar" ) )
